# Toward a Resident Personal Finance Curriculum: Quantifying Resident Financial Circumstances, Needs, and Interests

**DOI:** 10.7759/cureus.2540

**Published:** 2018-04-26

**Authors:** Ryan McKillip, Michael Ernst, James Ahn, Ara Tekian, Eric Shappell

**Affiliations:** 1 Pritzker School of Medicine, University of Chicago; 2 Section of Emergency Medicine, University of Chicago; 3 Department of Medical Education, University of Illinois at Chicago College of Medicine

**Keywords:** medical education, personal finance, resident finance education, resident wellness, curriculum development, needs assessment, educational loans, public service loan forgiveness

## Abstract

Introduction

Resident financial health has been linked to wellness and resiliency, yet financial literacy among residents is highly variable. While some medical school curricula include budgeting and student loan education, content on managing finances as a resident is usually lacking. We sought to quantitatively assess residents’ financial circumstances, needs, and interests to inform the design of a resident personal finance curriculum.

Methods

Surveys were sent to residents in eight specialties at an academic medical center. Likert-type responses allowed respondents to rate their level of comfort (1 = Very Uncomfortable, 7 = Very Comfortable) and interest (1 = Very Uninterested, 7 = Very Interested) in various personal finance topics including budgeting, loan repayment, disability insurance, life insurance, home buying, and retirement planning. Details regarding financial circumstances, including assets, liabilities, and insurance, were also collected. Results of questions that utilized a Likert-type scale are reported as median (interquartile range).

Results

Of 346 residents surveyed, 144 (41.6%) responded. Residents were from Internal Medicine (56, 38.9%), Pediatrics (34, 23.6%), Emergency Medicine (18, 12.5%), and other specialties (36, 25.0%). Ninety-one (63.2%) reported educational loans, with an average balance of $191,730. Credit card balances exceeding $3,000 were reported by 11 (7.6%) respondents. One-hundred-two (70.1%) reported emergency savings, but only 65 (45.1%) reported having a retirement account (average balance $27,608). Respondents rated highest comfort levels with budgeting (5[4–6]), and lowest level of comfort with disability insurance (2[2–4]) and home buying (2[2–5]). Interest in learning each topic was high (6[5–7]), with retirement planning (6[5–7]), investing (6[5–7]), and home buying (6[5–7]) the topics of highest interest.

Conclusion

These results highlight the deficits in personal finance literacy among residents. Future work should focus on development of a nationally scalable personal finance curriculum for residents.

## Introduction

Throughout the course of their training, residents frequently make important financial decisions with low financial literacy [1]. Misjudgments in loan management, home buying, and retirement planning can compound to significant repercussions later in life [2]. Despite evidence correlating financial health with physician wellness and resiliency [3-5], few residents report receiving financial planning education in residency, and financial preparedness is not emphasized within the Accreditation Council for Graduate Medical Education’s required competencies or milestones [6-8].

As the amount of outstanding education loans reported by graduating medical students continues to rise [9], deficits in resident financial literacy become more worrisome. Research from the United States Federal Reserve Board has found that families with education debt are more susceptible to financial distress [10]. Physicians are no exception; medical education debt and physician financial status are known to correlate with burnout and depressive symptoms [3]. In addition to its impact on personal life events such as when to start a family or buy a house [11], debt burden is consistently cited as a factor in career decisions such as medical student choice of specialty [12,13] and resident consideration of fellowships and entering into academic practice [14-16]. Small studies have already revealed the more immediate financial implications of educational loans and poor financial preparedness among residents [17-20]. Up to 20% of residents have outstanding credit card debt, 12% of residents have a credit card balance that exceeds $10,000, and only 39% of residents with children have started saving for college education [17-20]. This demonstrates the pressing need for providing residents with skills for navigating financial decision-making.

A nationally-scalable personal finance curriculum could help residents manage these challenges, however the appropriate content for such a curriculum is uncertain. Therefore, in an effort to develop a resident finance curriculum and follow Kern’s framework for the development of medical education curricula [21], we performed a targeted assessment of resident needs and interests regarding this subject. Building upon a qualitative study that explored the financial experiences and concerns of residents [22], this study attempts to quantitatively assess trainee sentiments in a broad, multi-specialty sample to determine which content areas are most essential to a resident personal finance curriculum.

## Materials and methods

Study design

An online anonymous survey format was used for this study. Survey content was informed by a recent qualitative study of resident finances [22], consistent with the instrument development model for mixed methods [23]. The survey was developed iteratively by faculty with formal training in survey research methodology and previous training in personal finance, and an author previously employed as a Certified Public Accountant in the financial services industry. Response process validity was established using Messick’s framework for assessment validation [24] by piloting the survey in the form of read-aloud sessions with a representative sample of three residents. Revisions were made according to feedback from the pilot and consisted of wording modifications for improved interpretation of questions. Likert-type responses allowed respondents to rate their level of comfort (1 = Very Uncomfortable to 7 = Very Comfortable), and interest (1 = Very Uninterested to 7 = Very Interested) in personal finance topics identified in the qualitative study [22]: budgeting, loan repayment, disability insurance, life insurance, home buying, and retirement planning. In addition, the survey asked residents to report details of their current household income, savings, debt, disability insurance, and life insurance. Household income was reported by selecting a range, which was based on the 2016 individual income tax brackets. Finally, demographics information was collected, and residents were asked to report whether they have received any previous education in personal finance in medical school or residency (e.g., lectures or course work). A copy of the survey is provided in the appendix.

Study setting and population

In May 2017, program directors at an urban academic medical center with over 1,000 residents and fellows were contacted to invite their respective residents to complete the anonymous survey. Program directors from eight specialties consented to have their residents contacted. Unique survey links were emailed to residents in June 2017. A reminder email was sent one week later. Residents were given three weeks to complete the survey. No compensation was offered for completing the survey. The surveys were collected and managed using Qualtrics software (Provo, UT). The University of Chicago Institutional Review Board granted exemption from review for this study.

Data analysis

Resident comfort and interest levels with each topic were quantified using the Likert-type responses. The topics of highest and lowest comfort and interest were identified. As a secondary analysis, using Wilcoxon Rank-Sum tests, we analyzed whether being from a primary care residency or having previous education in personal finance were associated with different comfort levels or interests. Surveys returned with no responses were excluded from analysis. Individual questions that were left blank were excluded from the analysis of that item or are reported as “No Response.” Results of questions that utilized a Likert-type scale are reported as median (interquartile range).

## Results

Of 346 residents surveyed, 144 (41.6%) submitted responses. Respondents were from Internal Medicine (56, 38.9%), Pediatrics (34, 23.6%), Emergency Medicine (18, 12.5%), and other specialties (36, 25.0%). A majority were postgraduate year 1 (PGY-1) (35, 24.3%) or PGY-2 (51, 35.4%), had an estimated household income in the $37,651 to $91,150 tax bracket (81, 56.3%), and reported no previous education in personal finance (99, 68.8%). The demographics of the residents who completed surveys are shown in Table 1.

**Table 1:** Characteristics of survey respondents. PGY: Postgraduate year

	N = 144	%
Gender		
Female	62	43.1
Male	65	45.1
No response	17	11.8
Age		
Mean (standard deviation)	29.1 (1.8)	
Marital status		
Married	56	38.9
Single	53	36.8
Living with partner	17	11.8
Divorced	1	0.7
Other	2	1.4
No response	15	10.4
Estimated household income		
$37,651 to $91,150	81	56.3
$91,151 to $190,150	37	25.7
$190,151 to $413,350	7	4.9
Not sure	3	2.1
Prefer not to answer	2	1.4
No response	14	9.7
Year of training		
PGY-1	35	24.3
PGY-2	51	35.4
PGY-3	34	23.6
PGY-4	8	5.6
No response	16	11.1
Specialty		
Internal medicine	56	38.9
Pediatrics	34	23.6
Emergency medicine	18	12.5
Psychiatry	10	6.9
Orthopedics	9	6.3
Obstetrics and gynecology	8	5.6
Neurology	7	4.9
Otolaryngology	2	1.4
Previous education in personal finance in medical school or residency (e.g., lectures or course work)		
Yes	45	31.2

Comfort and interests in personal finance topics

Respondents were uncomfortable with many personal finance topics, with the median overall response being “Slightly Uncomfortable” (3[2–5]). Topics with the lowest reported levels of comfort were disability insurance (2[2–4]), and home buying (2[2–5]). Additionally, the majority of respondents felt uncomfortable with the topics of life insurance (3[2–4]), investing (3[1–4]), and retirement planning (3[2–5]). Respondents reported the most comfort with regard to budgeting (5[4–6]), and comfort regarding loan repayment was approximately neutral (4[3–5]).

**Figure 1:**
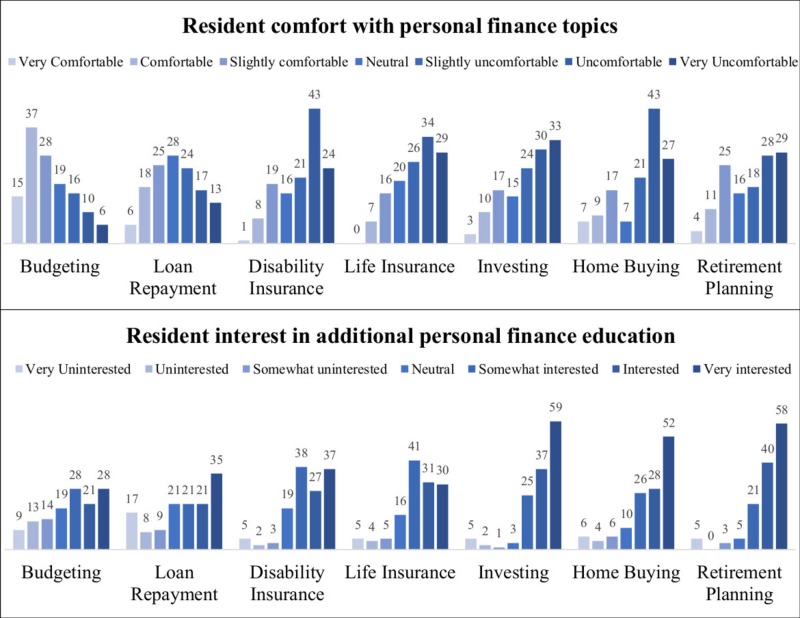
Respondent comfort levels and interests in personal finance topics.

Interest in learning more about each topic was high, with the median overall response being “Interested” (6[5–7]). Despite the reported comfort with budgeting, respondents were still “Slightly Interested” (5[3–6]) in learning more about this topic. The topics of highest interest to the respondents were retirement planning (6[5–7]), investing (6[5–7]), and home buying (6[5–7]). Respondents with educational loans were significantly more interested in learning about loan repayments (6[4.5–7] vs. 2[1–4], P < 0.01). The complete distribution of responses to the comfort and interest questions is presented in Figure 1.

While both groups expressed an overall discomfort with the personal finance topics, there were several differences between respondents who did and did not report receiving previous personal finance education. The 45 respondents with previous personal finance education were more comfortable in their present knowledge with regard to budgeting (5[4–6] vs. 5[3–6], P = 0.01), loan repayment (5[4–5] vs. 3[2–5], P < 0.01), disability insurance (3[2–5] vs. 2[2–4], P < 0.01), investing (3[2–5] vs. 2[1–4], P = 0.04), and retirement planning (4[3–5] vs. 2[1–5], P < 0.01). However, despite their differences in comfort, there were no statistically significant differences in the groups’ interest in learning more about each topic (Table 2).

**Table 2:** Comfort levels and interests among residents reporting previous education in personal finance. PF: Personal finance. Likert-type responses reported as median (interquartile-range). ^a^Previous education defined as either lectures or course work completed in medical school or residency.

	Comfort with personal finance topics			Interest in additional learning		
	No previous PF education	Previous PF education^a^		No previous PF education	Previous PF education^a^	
	N = 99	N = 45	P Value	N = 99	N = 45	P Value
Budgeting	5[3–6]	5[4–6]	0.014	5[4–6]	5[3–6]	0.372
Loan repayment	3[2–5]	5[4–5]	0.001	5[3–7]	5[4–​​​​​​​6]	0.825
Disability insurance	2[2–​​​​​​​4]	3[2–​​​​​​​5]	0.007	5[5–​​​​​​​7]	5[4–​​​​​​​7]	0.441
Life insurance	2[1–​​​​​​​4]	3[2–​​​​​​​4]	0.078	5[5–​​​​​​​6]	5[4–​​​​​​​7]	0.704
Investing	2[1–​​​​​​​4]	3[2–​​​​​​​5]	0.045	6[5–​​​​​​​7]	6[5–​​​​​​​7]	0.860
Home buying	2[2–​​​​​​​4]	2[2–​​​​​​​5]	0.823	6[5–​​​​​​​7]	6[5–​​​​​​​7]	0.381
Retirement planning	2[1–​​​​​​​5]	4[3–​​​​​​​5]	0.009	6[5–​​​​​​​7]	6[6–​​​​​​​7]	0.735

The 54 residents from specialty care residencies (Emergency Medicine, Psychiatry, Orthopedics, Obstetrics and Gynecology, Neurology, Otolaryngology) were more interested in learning about disability insurance than the 90 residents from primary care-based specialties (Internal Medicine, Pediatrics) (6[5–7] vs. 5[5–6], P < 0.01). There were no other statistically significant differences between specialty and primary care residents with regard to comfort or interest levels by topic.

Personal finance status and preparedness

Debts

Educational loans, held by 91 (63.2%) of respondents, were the most common form of debt, of which the average balance was $191,730 (median $200,000). Forty-seven (52.8%) owed at least $200,000. The number of residents with outstanding educational loans did not significantly vary with estimated household income, grouped by income tax brackets $37,651 to $91,150 (58, 75.3%), $91,151 to $190,150 (27, 73.0%), and $190,151 to $413,350 (3, 42.9%), (P = 0.18). The majority of respondents with educational loans felt at least “Slightly Comfortable” (5[3–5]) with current payoff plans. While less common, mortgages were reported by more than one in five respondents (29, 20.1%), and represented an average balance of $227,889 (median $200,000). Outstanding credit card loans were reported by 13 (9.0%) respondents, with 11 (7.6%) having a balance exceeding $3,000. Lastly, car loans were reported by eight (5.6%) respondents, with an average balance of $9,250. No other forms of debt were reported by the respondents.

Assets

A majority of respondents (102, 70.1%) reported having an emergency savings account with an average balance of $20,821 (median $10,000). Married respondents (51, 91.1%) were more likely than single respondents (31, 58.5%) to have an emergency savings account, P < 0.001. Respondents with emergency savings funds generally agreed with the statement “I am comfortable with the amount of emergency funds I have available” more than those who did not report having an emergency savings fund (6[5–6] vs. 2[1–4], P < 0.001).

**Figure 2:**
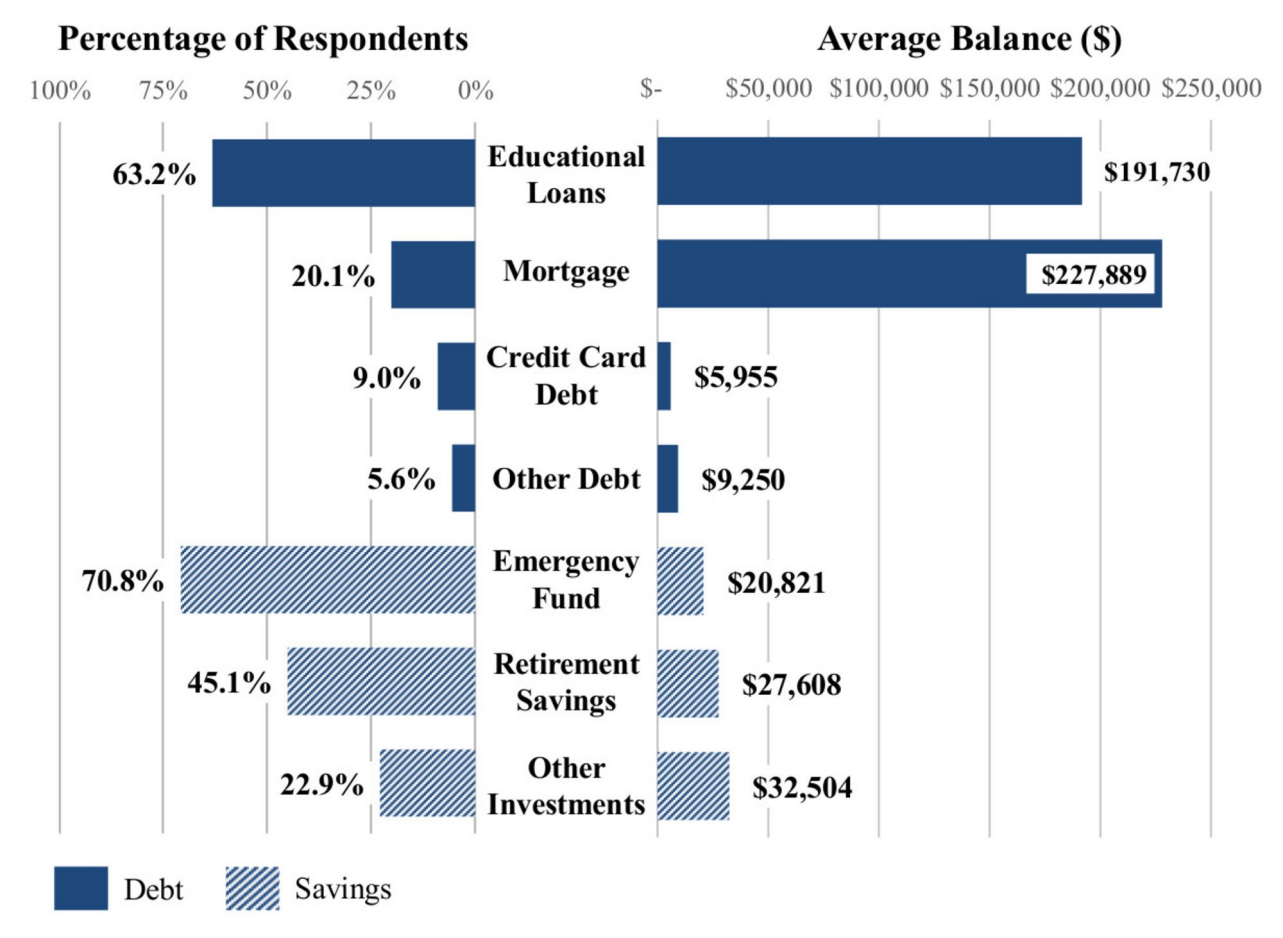
Respondent debt and savings positions.

Only 65 (45.1%) reported a retirement savings account, with an average balance of $27,608 (median $13,000). Other investments, averaging $32,504 (median $11,000), were reported by 33 (22.9%) respondents. Married and single respondents had similar rates of retirement accounts (31, 55.4% vs. 24, 45.3%, P = 0.391). Respondents generally disagreed with the statement “I am comfortable with the pace of my savings for retirement” (3[2–5]). Respondents with educational loans had less confidence in the status of their emergency funds (5[2–6] vs. 6[4–6], P = 0.03) and the pace of their retirement savings (3[2–4.5] vs. 4[2.5–6], P = 0.01) than residents with no educational loans. Twenty (13.9%) respondents denied having either an emergency fund or retirement savings account. A summary of debt and savings positions is presented in Figure 2.

Insurance

Just 56 (38.9%) of the respondents reported having disability insurance, and 25 (17.4%) were “Not Sure”. Similarly, 40 (27.8%) reported life insurance and 23 (16.0%) were “Not Sure”. Nearly half (63, 43.8%) denied having either form of insurance. When asked whether they agree with statements that they are comfortable with the process of selecting disability or life insurance, the majority of respondents disagreed (3[2–4] and 2[2–4], respectively).

Financial Advisors

Few respondents (25, 17.4%) reported having a financial advisor. The most common reasons cited for not having a financial advisor were cost (48, 45.7%), not knowing how to find one (34, 32.4%), and denying the need for one (31, 29.5%) (answers in this segment were non-exclusive). Eighteen respondents (17.1%) indicated they would not trust a financial advisor. Additionally, respondents overall disagreed (3[2–5]) with the statement “I feel comfortable finding reliable and unbiased financial advice.”

## Discussion

These data reflect overall deficits in personal finance literacy among residents, and demonstrate a strong interest in further learning on the subject. The majority of residents reported feeling uncomfortable with most topics and were interested in additional education on all surveyed topics. Residents who reported previous education in personal finance, whether through a course or lecture in either medical school or residency, were more comfortable with several of the personal finance topics. This observation is far from being concrete evidence of the effectiveness of these interventions, but shows an association with increased confidence. Additionally, despite higher comfort levels with the topics of budgeting, loan repayment, disability insurance, life insurance, investing, and retirement planning, those respondents had high interest in further education, suggesting that regardless of previous knowledge or experience, residents want more financial education.

A recently published study of resident personal finance literacy by Ahmad et al. utilized an objective knowledge assessment and surveyed satisfaction with financial condition and investment-risk tolerance [17]. When compared to our results, the 422 respondents in that study reported higher rates of student loans (71% vs. 63%), with comparable outstanding balances (48% owing at least $200,000, vs. 52% in our study). These balances are in line with the median educational loans reported on the 2016 Association of American Medical Colleges national survey of medical school graduates ($205,000) [9]. Rates of mortgage debt (32% vs. 20%) and retirement savings (62% vs. 45%) were also higher on the Ahmad study compared to ours. While these variances may reflect the economic differences in the practice settings of the institutions that were studied, among other variables, both studies reiterate the pattern of high debt burden and low financial literacy previously reported [9,20]. In addition to gathering additional data regarding residents’ financial circumstances, our study also reveals the varying levels of comfort that residents have with these topics and topic-level insight into the residents’ learning interests. Our data suggest that education covering investing, home buying and retirement planning will likely attract more attention from residents than budgeting and loan repayment. These data can inform curriculum design decisions and may aid in the development of wellness initiatives designed to address resident burnout.

Opinions regarding personal assets and liabilities provide additional insight for understanding the topic interests expressed by the respondents. A majority of the respondents in our study who had educational loans felt comfortable with their current plans for paying them off. This is despite loans well exceeding the national average and anxiety among residents regarding the prospect of discontinuation of the federal Public Service Loan Forgiveness program [22]. In contrast, respondents were uncomfortable with their current savings, their pace of savings for retirement, and the process of selecting disability and life insurance. Confidence regarding loan payoffs but apprehension about pace of retirement savings corresponds with our observation that residents were more interested in learning about retirement planning and insurance than budgeting and debt management. Residents may recognize that because they are early in their career, decisions about retirement planning and disability insurance can have important implications for future financial wellbeing. Furthermore, our data revealed that interest in learning about disability insurance was highest among residents from specialty care residencies, suggesting that these residents may be aware that a disability can have significant consequences for physicians in specialty care.

The pattern of low confidence with personal finance and strong interest in additional learning suggests that residents are aware of their knowledge deficits. Despite this awareness, fewer than one in five use a financial advisor. This may relate to the finding that the majority of residents lack confidence in their ability to find reliable and unbiased financial advice—a finding which may be related to the reputation of the financial services industry with regard to dishonest practices. Financial advisors consistently rank among the least trustworthy professionals, and up to 15% have been disciplined for selling inappropriate products (e.g., misrepresenting investments or annuities), charging aggressive fees, or other forms of misconduct [25]. Stronger personal finance education will allow residents to confidently make their own financial decisions, as well as sort legitimate and well-intentioned financial advising from predatory offers.

Limitations of this study include that the residents surveyed were from a single institution in an urban environment in the Midwest. Also, while a variety of specialties are represented in these data, this study did not include all specialties and most heavily sampled residents from three-year medical specialties (Internal Medicine, Pediatrics, and Emergency Medicine). Certain demographics such as family size, number of earners contributing to household income, and number of dependents were not collected. Survey answers did not specify whether they represent the individual respondent or household. These constraints introduce the possibility of coverage error and may affect the generalizability of our findings. Furthermore, there may be selection bias in the residents that chose to answer the survey. It is possible that there were different levels of experience and interest in the rest of the population with regard to finance. The loans reported in this study were those that had outstanding balances at the time of the survey; we did not collect information on loans previously paid or settled. We did not investigate whether residents’ educational institutions provided loan education or whether a financial expert was present during previous personal finance education. Finally, these data are self-reported and may suffer from recall error and/or rounding.

## Conclusions

This study provides objective corroboration of previous qualitative evidence that residents across specialties and years of training have high interest in further financial education, regardless of previous experience. The limited savings and significant debt burden carried by the residents in our study further necessitates an educational intervention that can enable them to make informed financial decisions. This study provides insight into the topics that are most interesting and important to residents, which can be used to inform the development of personal finance curricula for residency programs.

## Appendices

Resident Personal Finance Survey

Q1. Note to Participants: The authors acknowledge that this survey includes questions that some respondents may feel inquire about sensitive information. Several steps have been taken to ensure that individual-level data will not be identifiable and only group-level information will be reported. The authors wish to reiterate that participation in this survey is voluntary and that any or all questions may be skipped at any time.

Q2. Do you have any previous education in personal finance in medical school or residency (i.e., lectures or course work)?

Yes (1)

No (2)

Q3. Please briefly describe your previous personal finance education:

Q4. Please rate your level of comfort with your knowledge on the following topics: 

Very uncomfortable (1); Uncomfortable (2); Slightly uncomfortable (3); Neutral (4); Slightly comfortable (5); Comfortable (6); Very comfortable (7)

Budgeting

Loan repayment options

Disability insurance

Life insurance

Investing

Home buying

Retirement planning

Q6. Please rate your level of interest in learning about the following topics: 

Very uninterested (1); Uninterested (2); Somewhat uninterested (3); Neutral (4); Somewhat interested (5); Interested (6); Very interested (7)

Budgeting 

Loan repayment options 

Disability insurance 

Life insurance 

Investing 

Home buying 

Retirement planning 

Q7. Please rate your level of agreement with the following statements:

Strongly disagree (1); Disagree (2); Somewhat disagree (3); Neutral (4); Somewhat agree (5); Agree (6); Strongly agree (7)

I am comfortable with the amount of emergency funds I have available

I am comfortable with the pace of my savings for retirement

I am comfortable with the process of selecting disability insurance

I am comfortable with the process of selecting life insurance

I am comfortable with the process of buying a home

I am comfortable finding reliable and unbiased financial advice

I am comfortable managing my personal finances on my own

I am comfortable with my current plans for paying off loans

Q9. Do you have education debt?

Yes (1)

No (2)

Q10. Approximately how much education debt do you have? (please use only numbers)

Q11. Do you have credit card debt (i.e., a balance that is not paid in full each month)?

Yes (1)

No (2)

Q12. Approximately how much credit card debt do you have? (please use only numbers)

Q13. Do you have a mortgage?

Yes (1)

No (2)

Q14. Approximately how much is your mortgage debt? (please use only numbers)

Q15. Do you have other sources of debt?

Yes (1)

No (2)

Q16. What other sources of debt do you have? (please answer in text)

Q17. Approximately how much debt do you have from each source listed above? (please assign an approximate number to each source)

Q18. Do you have an emergency fund saved? (this may include funds in checking and/or savings accounts but does not include retirement accounts)

Yes (1)

No (2)

Q19. Approximately how much do you have in your emergency fund? (please use only numbers)

Q20. Do you have a retirement savings account?

Yes (1)

No (2)

Q21. What kind(s) of retirement accounts do you have?

401(k) (1)

403(b) (2)

IRA (3)

Roth IRA (4)

Other (5)

Q22. What other types of retirement accounts do you have?

Q23. Approximately how much do you have saved in retirement accounts? (please use only numbers)

Q24. Do you have an investment account outside of retirement accounts (i.e., a taxable investment account)?

Yes (1)

No (2)

Q25. Approximately how much do you have saved in your non-retirement (i.e., taxable) investment accounts? (please use only numbers)

Q26. Do you have disability insurance?

Yes (1)

No (2)

Not sure (3)

Q27. What kind of disability insurance policy do you own?

Group policy (1)

Individual policy (2)

Not sure (3)

Q29. Do you have life insurance?

Yes (1)

No (2)

Not sure (3)

Q30. What kind(s) of life insurance policies do you have?

Term (1)

Permanent (including whole or universal) (2)

Not sure (3)

Q31. Approximately how much life insurance do you own?

Less than $250,000 (1)

$250,001 to $500,000 (2)

$500,001 to $750,000 (3)

$750,001 to $1,000,000 (4)

More than $1,000,000 (5)

Not sure (6)

Q32. Do you have a professional financial advisor?

Yes (1)

No (2)

Q33. Please mark the reasons you do not have a financial advisor

I don’t think I need a professional financial advisor (1)

I don’t know how to find a professional financial advisor (2)

I wouldn’t trust a professional financial advisor (3)

A professional financial advisor would be too expensive (4)

Other (5)

Q34. Please describe the reason(s) you do not have a financial advisor

Q35. Please estimate your household income (figures represent 2016 tax brackets)

Less than $37,650 (1)

$37,651 to $91,150 (2)

$91,151 to $190,150 (3)

More than $190,150 (4)

Not sure (5)

Prefer not to answer (6)

Q36. Age

Q37. Marital status

Single (1)

Living with partner (2)

Married (3)

Divorced (4)

Other (5)

Prefer not to answer (6)

Q38. Please describe your marital status

Q39. Training level

PGY-1 (1)

PGY-2 (2)

PGY-3 (3)

PGY-4 (4)

PGY-5 (5)

PGY-6 or greater (6)
